# Unusual Presentation of a Rare Tumor of the Dorsal Surface of the Foot

**DOI:** 10.5402/2011/704549

**Published:** 2011-03-31

**Authors:** O. Hocar, H. Yacoubi, N. Akhdari, S. Amal, F. Ait Essi, M. Latifi, H. Rais, B. Belaabidia

**Affiliations:** ^1^Dermatology Department, School of Medicine, Cadi Ayyad University, Mohammed the VIth University Hospital, Marrakesh 40000, Morocco; ^2^Trauma and Orthopaedic Department, School of Medicine, Cadi Ayyad University, Mohammed the VIth University Hospital, Marrakesh 40000, Morocco; ^3^Pathology Department, School of Medicine, Cadi Ayyad University, Mohammed the VIth University Hospital, Marrakesh 40000, Morocco

## Abstract

Calcifying aponeurotic fibroma (CAF) was originally described by Keasbey in 1953 as juvenile aponeurotic fibroma, most commonly occurring in the palms of the hands and soles of the feet in children and adolescents. It usually presents as a firm, painless mass without preceding trauma. We report a case of this rare condition with an unusual presentation in a 60-year-old woman affecting the dorsal surface of the foot. It is a relatively benign condition with a good prognosis following complete surgical excision. It may have a slightly increased incidence in males. The accurate diagnosis is based only on histology but it is essential to differentiate it from other sinister lesions such as fibrosarcoma that may lead to amputation.

## 1. Introduction

Calcifying aponeurotic fibroma (CAF) is a rare, locally aggressive fibroblastic lesion occurring primarily in the palms of the hands and soles of the feet in young children and adolescents under 20 years of age [1]. Clinically and radiologically it is similar to other more common and sinister lesions requiring radical surgery. Due to its rare occurrence, there are only a few reported cases in the dermatologic literature. The present report discusses an aponeurotic calcifying fibroma of the dorsal surface of the foot in a 60-year-old woman and reviews the current literature on this rare entity.

## 2. Case Report

**Figure 1:**
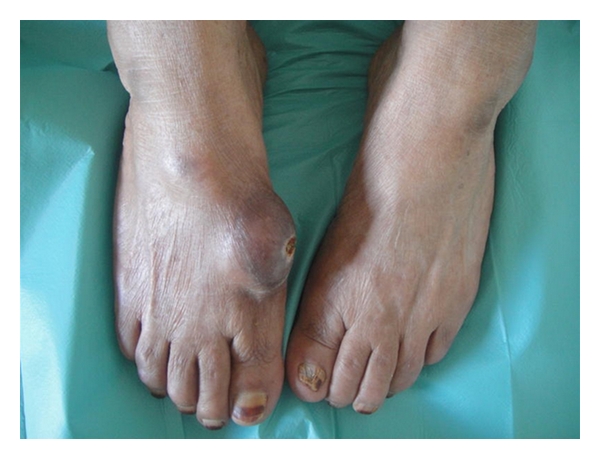
Large mass on the dorsal surface of the right foot of a 60 year-old woman.

A 60-year-old woman in good general condition with no history of any trauma exhibited a palpable mass on the dorsal surface of the right foot. The lesion slowly enlarged over 12 years. Physical examination revealed a 4.5 × 4.5 cm, hard, painless, immobile mass; it was well defined, round with ulceration at the end (Figure 1(a)). Radiographs of the right foot revealed a calcifying soft tissue mass without bone involvement (Figure 1(b)). A lesion biopsy was performed and histological exam showed nodular deposits of calcification with chondroid differentiation, each surrounded by a palisade of rounded, chondrocyte-like cells, between the coalescent calcified nodules and emanating into the surrounding soft tissues without pleomorphism, atypia, or mitotic activity, the stroma of nodules was hyalinised (Figure 1(c)). Diagnosis of calcifying aponeurotic fibroma was established. Total excision of the lesion was performed and patient had an uneventful postoperative recovery. During the last six months, the patient has been well with no signs of recurrence.

## 3. Discussion

 Calcifying aponeurotic fibroma (CAF) was first described by Keasbey in 1953 as “juvenile aponeurotic fibroma” occurring in the palms and soles of the children [1]. Although less well known, CAF can also occur in a wide variety of other less common locations. A review of the literature shows 45 cases affecting unusual sites such as the back, forearm, knee region, and thigh. In our knowledge, our case is the first one with a CAF in the dorsal surface of the foot. It seems to have a male predominance, particularly in children and young adults, with a peak incidence at ages 8–14 years [2, 3]. The aetiology of the tumour is uncertain [1]. The lesion typically ranges in size from 1 to 5 cm, and is often present for years before removal, owing to its indolent growth characteristics [2]. Radiologically, CAF may show a soft tissue mass with no associated osseous lesions and a fine stippling of focal calcification [4]. However, in extremely rare cases, occasional scalloping of the cortex [5, 6] and thickening of the bone [7] have been reported in pediatric patients.

Clinical features such as the patient's age, site of lesion and calcific stippling on plain radiographs are suggestive of CAF but they are not diagnostic; several conditions share similar clinical features including infantile and juvenile forms of fibromatosis, a fibrous hamartoma of infancy, the monophasic fibrous subtype of synovial sarcoma, and a chondroma of soft parts.

Histologically, CAF is comprised of varying degrees of fibrous connective tissue arranged in a fascicular pattern. The lesion is bland in appearance and composed of plump spindle cells with round to ovoid nuclei with indistinct cell borders. The nuclei may be arranged in a palisading fashion. Extension of the fibrous connective tissue into the adjacent surrounding tissue is not uncommon [1]. In addition, perivascular and perineuronal proliferations may arise within the stroma. Mitotic activity is not common, and cellular atypia is not observed. All authors have reported areas of calcium deposition, which do not occur in areas of degeneration. Two phases in the tumor's development have been described [3]: an initial phase, more common in infants and young children, in which the tumor grows diffusely, and a late phase, in which the lesion is more compact and nodular. Calcification and cartilage formation may be identified in the latter phase of CAF development [1, 2]. The calcified component has been described as fine granules or large amorphous masses. Mature bone with hematopoietic elements is noted in some cases of CAF, representing an unusual form of mineralization which may explain the longstanding history of the lesion [8]. In addition, CAF may exhibit chondroid differentiation, with epitheliod fibroblasts and osteoclast-type giant cells [2]. In the present lesion, distinct cartilage differentiation was observed. The term *aponeurotic *has been used because there is a subtle transition from fibrous connective tissue to fibrocartilage as tendons, aponeuroses, and ligaments insert into bone through Sharpey fibers, and the location of many of these tumors suggests an aponeurotic origin [9]. Malignant transformation is very rare [10]. Given a local recurrence rate of approximately 50%, complete excision with a margin of normal tissue is advisable when this can be accomplished without functional compromise. In view of its benign nature and good prognosis after resection, the treatment of choice is local surgical excision.

Recurrence is common especially in infants and children. However, local re-excision can be performed with a favourable prognosis.

In summary, CAF is a relatively rare benign soft tissue tumour occurring in children and young adults. It usually presents as a firm, painless mass in the soles and hands without preceding trauma. It may have a slightly increased incidence in males. The accurate diagnosis is based only on histology but it is essential to differentiate it from other sinister lesions such as fibrosarcoma that may lead to amputation. In our case, the presentation was unusual as the lesion appeared to develop in the dorsal surface of the foot and occured in a 60-year-old female.

##  Conflict of Interest

None identified.

## Figures and Tables

**Figure 2:**
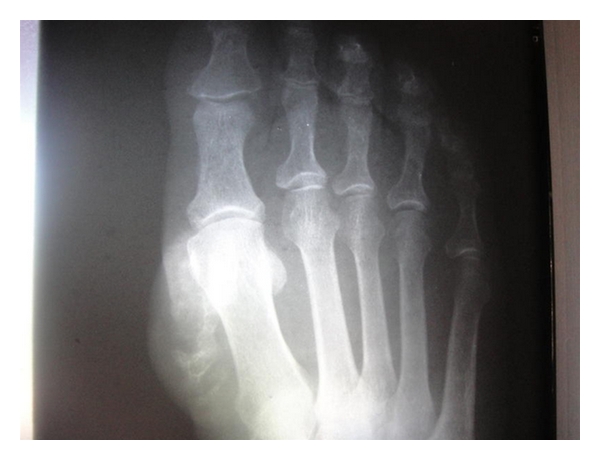
Radiograph showing soft tissue mass with calcification.

**Figure 3:**
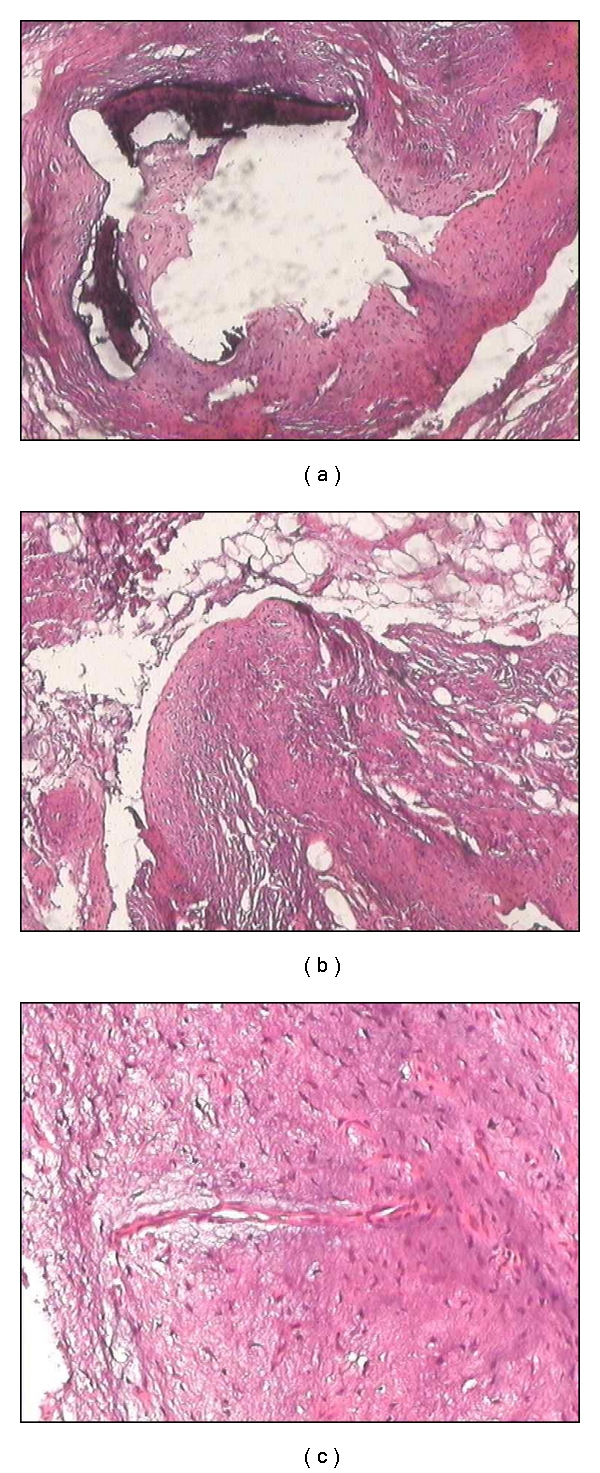
Histopathology of lesion representing nodular deposits of calcification, each surrounded by a palisade of rounded, chondrocyte-like cells (hematoxylin and eosin ×200).
